# Assessment of Psychiatric Symptoms Using SCL-90-R among HIV/AIDS Individuals in Razavi Khorasan Province, Iran

**Published:** 2011-01-01

**Authors:** M R Hedayati-Moghaddam, I Eftekharzadeh Mashhadi, R Zibaee, A M Hosseinpoor, F Fathi-Moghaddam, H Bidkhori

**Affiliations:** 1 Research Center for HIV/AIDS and Viral Hepatitis,Iranian Academic Center for Education, Cultureand Research (ACECR), Mashhad Branch, Mashhad, Iran; 2 Health Center of Razavi Khorasan Province, Mashhad, Iran

**Keywords:** Psychiatric symptoms, HIV/AIDS, Mashhad, Iran

Dear Editor,

HIV infection, the same as other chronic diseases, is associated with psychiatric symptoms.[1] A profound psychological distress is made after the diagnosis of infection, and several psychiatric disorders including AIDS phobia, bereavement and grief, anxiety and depression were shown as a reaction to the infection.[2] Associating the infection to the stigmatized and illegal behaviors by the family, friends, and others, especially in the developing countries, as well as the nature of the infection that is frustrating and incurable, both affect the patients enormously.[3] A lack in investigations on the psychiatric symptomatology of HIV infection in our country, which surely affects the patient’s compliance and management process, convinced us to assess the frequency and severity of psychiatric symptoms in those living with HIV in Razavi Khorasan Province, northeast of Iran.

From the 320 cases of HIV infection, documented in the Health Center of Razavi Khorasan Province, Mashhad, Iran, up to 2006, all above 15 years old with an ongoing management (n=61), were included in the study. Others were not accessible because of death, migration and non-cooperation. Demographic characteristics, clinical and laboratory findings were defined according to their management files. Symptom Checklist 90-Revised (SCL-90-R) with sensitivity and specificity of 80.9% and 92.7% in a national study,[4] was filled out by a clinical psychologist. The 90 items in the questionnaire were scored on a five-point Likert scale (0-4), indicating the rate of occurrence of the symptom during the time reference. The response rate was 82% (50/61). Respondents were 21-51 (35.9 ± 7.1) years old, 90% male, 74% married at least once, 52% unemployed, 46% with utmost 5 years education, 80% IUDs, 84% with a history of imprisonment, 30% AIDS patients, 66% HCV-Ab positive, and 12% HBs-Ag positive.

The checklist-derived scores were: Overall score 145.1±61.2 (42-305); GSI (Global Severity Index, average score of the 90 items) 1.6±0.68 (0.47-3.4), PST (Positive Symptoms Total, the number of items scored above zero) 60.9±16.9 (15-90), and PSDI (Positive Symptom Distress Index, the average score of the items scored above zero) 2.3±0.53 (1.2-3.4). The GSI is suggested to be the best single indicator of the current level of the disorder. Those with a GSI above 1 were considered to have psychiatric symptoms (79.6%). With the cut-off point of 0.7, defined in another study in Iran,[8] symptom prevalence was 85.7%. Severity of psychiatric symptoms according to the GSI index was low (1-1.99) in 46.9%, moderate (2-2.99) in 30.6%, and severe (≥3) in 2% of cases. Another study in Iran showed the frequency of psychiatric symptoms as high as 93.2%, among HIV-infected individuals using SRQ-24 test.[5] Although no study was found comparing these symptoms between HIV infected individuals and general population in Iran, frequency of these symptoms seems to be considerably higher, regarding to the results of population based studies.[6] In other countries, psychiatric symptoms were also highly frequent among HIV-infected individuals.[1][7]

The scores and symptom distress indices (PSDI) of each symptom and the related frequency was shown in Table 1. Except phobic anxiety with a 28 % frequency, others had a considerable frequency, ranging from 67-86%. Depression, obsessive compulsive and interpersonal sensitivity were the subscales with the highest frequency. Depression and anxiety were also the most frequent psychiatric symptoms in the other studies.[8][9] However, there is evidence that only symptomatic HIV disease, not HIV infection itself, increases the risk of major depression.[9]

**Table 1:** Scores and symptom distress indices, and frequency of each subscale in the SCL-90-R

**Subscale a**	**Score**	**PSDI**	**Frequency (%)**
Somatization [12]	21.5±11.2	1.8±0.93	38 (76)
Obsessive compulsive [10]	17.9±7.3	1.8±0.73	43 (86)
Interpersonal sensitivity [9]	14±6.8	1.6±0.75	40 (80)
Depression [13]	26.2±11.5	2±0.89	43 (86)
Anxiety [10]	16.5±8.5	1.7±0.85	39 (78)
Hostility [6]	9.8±6.5	1.6±1.1	35 (70)
Phobic anxiety [7]	4.8±4.1	0.7±0.59	14 (28)
Paranoid ideation [6]	9.2±4.9	1.6±0.81	37 (74)
Psychoticism [10]	12.6±6.7	1.3±0.67	34 (68)

^a^ Figure in the parenthesis is indicative of the number of questions in the respective subscale

In this study, HIV-positive cases were mostly male, middle-aged, low-educated and unemployed individuals. Low socio-economical status was reported in some studies to have significant association with psychological symptoms in HIV infection.[8] In addition, high prevalence of psychiatric disorders among prisoners and drug users has been showen in several studies. Furthermore, it was demonstrated that drug abuse could exacerbate the severity of mental distress in HIV-positive patients.[10] This could be another important factor that explains the considerable frequency of the symptoms among participants in our study with a prominent history of prison and injective drug use.

The high frequency of psychiatric symptoms, shown in this study, necessitates a precise attention to this aspect of HIV infection for an effective management of the HIV positive individuals.
